# Case report: Rare case of a preoperatively diagnosed spermatic cord paraganglioma and literature review

**DOI:** 10.3389/fonc.2024.1373727

**Published:** 2024-04-11

**Authors:** Yining Hao, Xiuci Li, Jing Xie, Wei He, Chenghe Wang, Fukang Sun

**Affiliations:** ^1^ Department of Urology, Ruijin Hospital, Shanghai Jiao Tong University School of Medicine, Shanghai, China; ^2^ Department of Pathology, Ruijin Hospital, Shanghai Jiao Tong University School of Medicine, Shanghai, China

**Keywords:** paraganglioma, spermatic cord mass, preoperative diagnose, neuroendocrine tumors (NET), urogenital system

## Abstract

Paraganglioma (PGL) is rare, and PGL that arises from the urogenital system is even rarer. Here we report a case of PGL in spermatic cord and review the relevant literatures. We encountered a 15-year-old boy with a history of hypertension for almost 2 years, accompanied with headache and palpitations. His serum and urine catecholamines were elevated, but no adrenal lesions were detected, suggesting the existence of PGL. Upon physical examination, a painless nodule adherent to the spermatic cord in the right scrotum was found. A systemic Ga^68^ DOTATATE PET-CT was then performed, and it revealed a mass with high DOTATATE uptake in the right scrotum. The CT, MRI, and ultrasound images showed the abundant blood supply to the tumor. Based on the above-mentioned imaging and biochemical information, a diagnosis of PGL was made prior to surgery. After 2 weeks of preparation with Cardura, an open surgery was performed to remove the tumor together with the right testis and right epididymis. The blood pressure increased to 180/100 mmHg when the tumor was touched intraoperatively and decreased to 90/55 mmHg after the tumor was removed. Post-operative pathology confirmed our diagnosis of PGL originating from the spermatic cord. Immunohistochemical (IHC) staining showed SDHB (+), CgA (+), synaptophysin (+), GATA3 (+), CD56 (+), sertoli cells S-100 (+), and Ki67 (5%). Genetic testing revealed a missense mutation in the SDHA gene. Only 16 cases of spermatic cord PGL have been reported to date. Although it is easy to diagnose by histology and IHC examinations, preoperative diagnosis is quite important as it can actually reduce intraoperative complications.

## Introduction

Paraganglioma (PGL) is a rare neuroendocrine tumor with an incidence of only 6.95 cases per million people (1). PGLs secrete norepinephrine (NE), epinephrine (E), dopamine (DA) and other catecholamines (CA), causing hypertension and other clinical symptoms (2). The location of primary PGL is diverse, including the head, neck and retroperitoneum. Here we report a case of PGL in the spermatic cord of a 15-year-old boy with a history of unexplained hypertension for almost 2 years.

## Case description

### Clinical findings

A 15-year-old boy was incidentally found to have hypertension (with a maximum blood pressure of 160/120 mmHg) during a physical examination in October 2021 and was admitted to a local hospital in July 2022. He felt occasional dizziness after an activity and had no fever, chills, fatigue, nausea, or vomiting. No family history of related diseases was detected. After a 24-h ambulatory blood pressure monitoring, he was treated with metoprolol and lercanidipine then switched to the combination of bisoprolol fumarate and fosinopril sodium.

**Figure 1:**
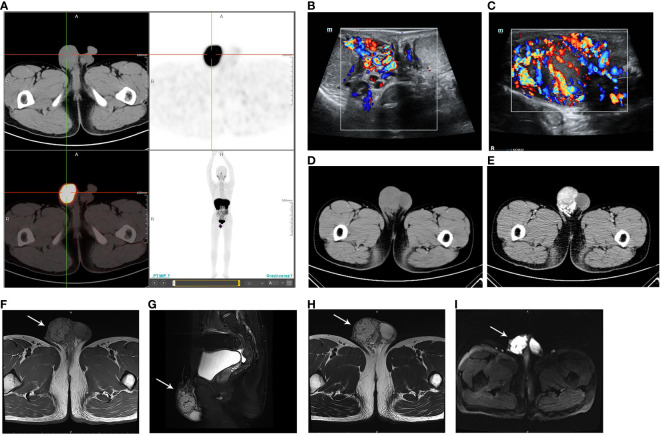
**(A)** Ga^68^ DOTATATE PET-CT showing a mass in the right scrotum, with a SUVmax value of 26.7 and a size of 38 × 47 mm. The DOTATATE uptake of the mass is significantly increased. **(B)** Scrotum ultrasound confirming a mass in the right tunica vaginalis cavity **(C)** with abundant blood flow signals. **(D)** Testicular CT demonstrating a low-density mass in the right scrotum. **(E)** The mass is obviously enhanced in contrast-enhanced imaging. **(F)** MRI revealing the low T1-weighted signal of the lesion. **(G)** Sagittal plane and **(H)** transverse section MRI images showing the high T2-weighted signal of the lesion. **(I)** Lesion presenting as hyperintense on diffusion-weighted imaging.

In March 2023, the patient was transferred to our hospital because of poor blood pressure control. He had paroxysmal headaches and palpitations. Upon physical examination, a painless nodule measuring 4 cm in diameter was found in the right scrotum, adherent to the spermatic cord. Other than that, there were no other positive clinical signs. No abnormalities were found in aldosterone level, renin level, aldosterone-to-renin ratio, serum and urine cortisol levels, thyroid function, routine urinalysis, urinary albumin to creatinine ratio, glomerular filtration rate, renal ultrasound, renal artery ultrasound, and renal artery computed tomography angiography. All of the above-mentioned procedures ruled out primary aldosteronism, Cushing’s syndrome, hyperthyroidism, renal hypertension, and renovascular hypertension. However, his serum and urine catecholamine levels were significantly elevated: serum NE 2,405.4↑ pg/mL (reference range: 19–121 pg/mL), urine free NE 1,003.13↑ ug/24 h (reference range: 7–65 ug/24 h), urine free E 28.04↑ ug/24 h (reference range: 0–22 ug/24 h), and urine free DA 445.45↑ ug/24 h (reference range: 75–440 ug/24 h), suggesting a possible PGL. Next, systemic Ga^68^ DOTATATE positron emission tomography-computed tomography (PET-CT) scan was performed, and it revealed a soft tissue mass in the right scrotum measuring approximately 38×47 mm, with a SUVmax value of 26.7, and the SUVmax ratio of this mass to the liver was 3.42 (**Figure 1A**). A suspicious lymph node metastatic focus was also detected in the left iliac lymph node region with a diameter of 6 mm, SUVmax value of 6.8, and the SUVmax ratio of lymph node to the liver was 0.87. No other abnormalities were found, and the SUVmax of the liver is 7.8. In addition, the scrotum ultrasound showed a 42.0 × 22.5 × 39.0-mm solid mass in the right tunica vaginalis cavity, with abundant blood flow signals (**Figures 1B, C**). Testicular enhanced computed tomography (CT) confirmed a low-density focus of 4.5 × 3.4 cm in the right scrotum (**Figure 1D**), and no significantly enlarged lymph nodes were seen in the bilateral groin or pelvis. After enhancement, the focus was obviously enhanced, and the blood supply was very abundant (**Figure 1E**). Pelvic enhanced magnetic resonance imaging (MRI) scan was further performed, and a 4.5 × 3.6-cm hypervascular lesion with abnormal signal in the right scrotum was revealed. The lesion had a low T1-weighted (T1w) signal and a high T2-weighted (T2w) signal, and it was hyperintense on diffusion-weighted imaging and exhibited a reduced signal on the apparent diffusion coefficient map (**Figures 1F–I**). Continuous enhancement of the lesion was observed on contrast-enhanced imaging. There was no evidence of enlarged lymph nodes. The above-mentioned imaging findings suggested the possibility of a neuroendocrine tumor that was inseparable from the right spermatic cord. In addition, he had no hereditary syndrome. To sum up, we preoperatively diagnosed his lesion as sporadic spermatic cord PGL.

### Surgery

On April 12, 2023, after completion of the relevant investigations and preoperative preparation, unilateral orchiectomy (right) and surgical resection of the right scrotum mass were performed under general anesthesia and invasive arterial blood pressure monitoring. Exploration of the right scrotum revealed a tumor with a diameter of about 4.5 cm. The tumor was inseparable from the right spermatic cord, and the blood vessels on the tumor’s surface were severely engorged. Palpation of the tumor significantly elevated the blood pressure to 180/100 mmHg. Thus, the tumor could not be resected alone. Through a close cooperation between the anesthesiologists and surgeons, the tumor was removed along with the right testis, epididymis, and spermatic cord, and the blood pressure dropped to 90/55 mmHg at last.

There were no complications that occurred, and the patient was discharged 2 days after the surgery. Then, the blood and urine catecholamine levels and blood pressure returned to normal ranges. Upon pelvic enhanced CT re-examination, no evidence of local recurrence or distant metastasis at 4 and 8 months after the operation was shown (**Supplementary Figure S1**). The serum NE levels at 2, 4, and 8 months after the operation were 91, 81, and 89.5 pg/mL, respectively.

### Pathological and genetic findings

**Figure 2:**
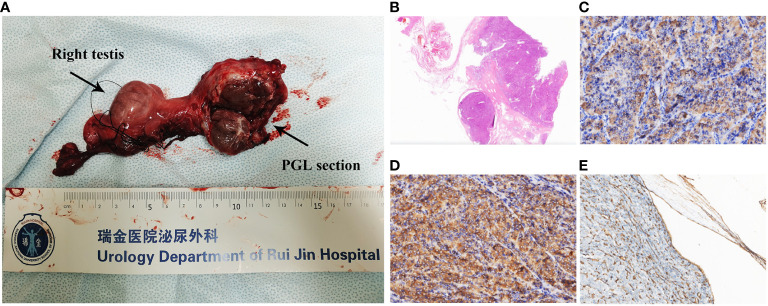
**(A)** Right spermatic cord, testis, epididymis, and tumor. The tumor is closely related to the spermatic cord, and its section is grayish yellow, solid, and with moderate hardness. A negative resection margin was obtained. **(B)** H&E staining (×4) of the tumor. Immunohistochemical staining (×200) of the tumor showing the **(C)** CgA positive, **(D)** SDHB positive, and **(E)** CD34 positive in intravascular tumor thrombus.

Pathological examination revealed a 4.0 × 4.0 × 2.5-cm mass in the spermatic cord to be PGL, with extraperitoneal invasion and endovascular tumor embolus. The section is grayish yellow, solid, and with moderate hardness; a negative resection margin was obtained (**Figure 2A**). No obvious abnormality was found in the testis, epididymis, or vas deferens. In hematoxylin–eosin (HE, **Figure 2B**) staining, the tumor cells were arranged in a pattern of nests with nuclear atypia. Immunohistochemical (IHC) staining depicted positive staining of chromogranin A (CgA, **Figure 2C**), succinate dehydrogenase (SDH) complex iron sulfur subunit B (SDHB, **Figure 2D**), CD34 (positive in blood vessels, **Figure 2E**), synaptophysin, S-100 (positive in sertoli cells), GATA binding protein 3 (GATA3), CD56 and Ki67 (5%). The IHC and morphology findings supported the diagnosis of spermatic cord PGL.

**Figure 3:**
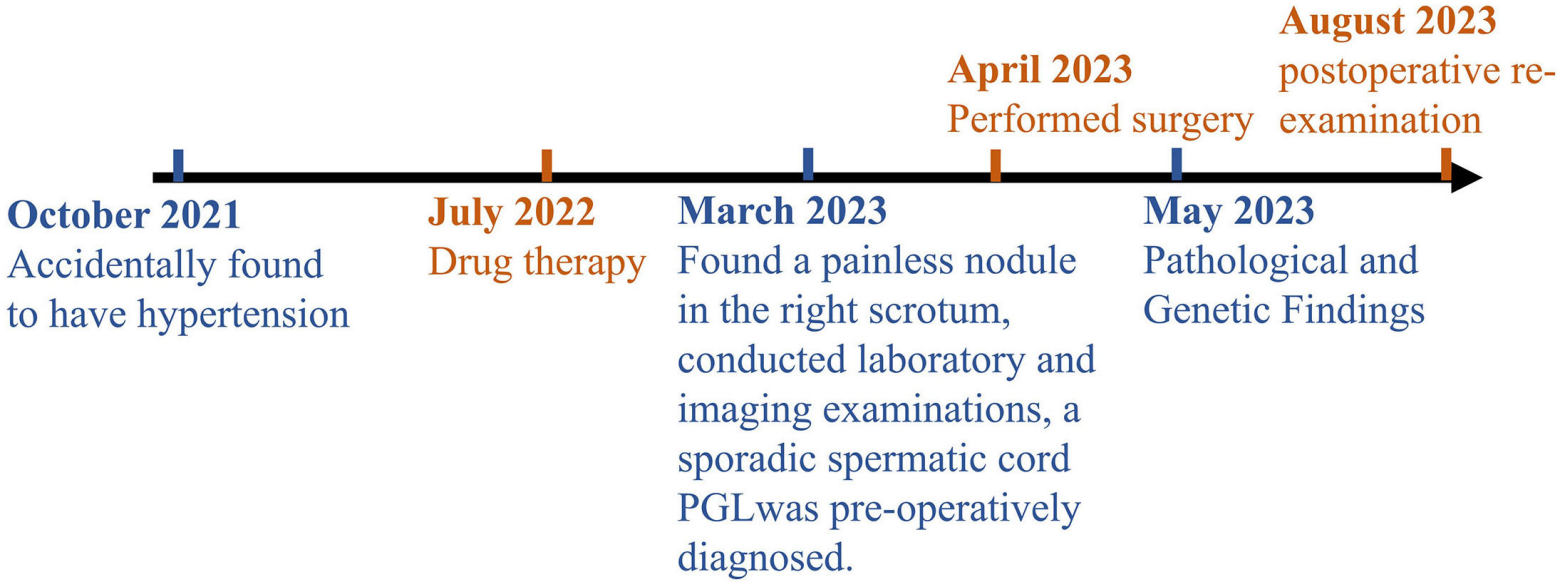
Timeline with relevant data from the episode of care.

To further investigate the genetic characteristics, an endocrine tumor panel analysis was performed. Among 46 candidate genes (**Supplementary Table S1**), we identified the c.C1334>T (p.Ser445Leu) succinate dehydrogenase complex flavoprotein subunit A (SDHA) missense mutation, with a mutation abundance of 0.773. The timeline with relevant data of this case is shown in **Figure 3**.

## Discussion

Pheochromocytoma and paraganglioma (PPGL) is a very rare neuroendocrine tumor, with an incidence of 0.4–9.5 per 1 million people and approximately 0.1% in patients with hypertension (3). PPGLs can be divided into pheochromocytoma (PHEO) and PGL according to their location. PHEOs originate in the adrenal medulla, and most are unilateral. In contrast, PGLs, which account for 15%–20% of PPGLs, occur outside the adrenal gland, such as in the head, neck, thorax, abdomen, retroperitoneum, and pelvis, and some lesions may be multiple (4). More than 50% of PPGL patients were undiagnosed (5), and persistent CA overproduction is fatal to the cardiovascular system, emphasizing the need for early diagnosis and treatment of PPGL (6).

Since the 2017 World Health Organization (WHO) classification of endocrine tumors, all PPGLs are considered as malignant neoplasms with metastatic potential (7). The 5-year overall survival rate of metastatic PPGL ranges from 34% to 60%. Those short-term survivors are patients with liver or lung metastasis, while long-term survival is observed in patients with bone metastasis (8). Although sporadic, 30% of PPGLs are considered as hereditary diseases and associated with germ line mutations (9). There are 22 definite susceptibility genes of PPGL, which can be categorized into a pseudohypoxic-related cluster and a kinase signaling-related cluster (10). Among them, mutations in SDH subunit genes (SDHA, SDHB, SDHC, SDHD, and SDHAF2) are the most common gene mutations in metastatic PPGL, and more than half of metastatic PPGL patients showed SDHx mutation (11, 12). SDHB mutation is associated with metastatic lesions and worse survival (13). Moreover, tumors in patients with SDHB and SDHD mutations cause familial PGL, which mainly produces dopamine (14).

The most common symptom of PPGL is hypertension. Patients may present with paroxysmal hypertension or permanent hypertension depending on the secretion rhythm of CA. Headache, palpitations, and hyperhidrosis are the typical triad of PPGL. They are caused by extra NE, E, and DA that are released from neoplastic chromaffin cells (2). Currently, the 24-h urinary free CA is the primary biochemistry test method for the qualitative diagnosis of PPGL, with a sensitivity of 84% and a specificity of 81% (15). However, CA is secreted by chromaffin cells discontinuously, while their metabolism is continuous. Therefore, the metabolite metanephrines (MNs) have higher diagnostic sensitivity (16, 17). The localization diagnosis includes anatomical imaging (CT, MRI, or ultrasound) and functional imaging (18). CT is the first choice for screening PPGL. The contrast-enhanced CT has a sensitivity of nearly 100% and a specificity of about 80%, which reveals the morphology of the tumor and has a spatial resolution (19). MRI is also recommended and is suitable for patients with contrast media allergy as well as for children and pregnant women. The sensitivity of MRI is similar to that of CT, and it performs better in evaluating the vascular invasion, recurrent, or metastatic lesions (4). Ultrasound is an optional method, which is simple, non-invasive, and convenient, but its sensitivity is lower than CT and MRI, and it cannot be used for positioning. DOTATATE and FDG PET-CT have high diagnostic accuracy and are more informative than CT and MRI for metastatic diseases (20–22). Due to the high DOTATATE uptake by PPGL cells, DOTATATE PET-CT has a better tumor contrast than FDG PET-CT, which allows for diagnosis or exclusion of a disease (20). In this case, the CA levels of this patient were obviously increased, and serum NE was higher than three times of the critical value, suggesting the existence of PPGL. As no adrenal lesions were detected, Ga^68^ DOTATATE PET-CT was performed, and it revealed a neoplasm with DOTATATE uptake in the right spermatic cord. Subsequent MRI and ultrasound further demonstrated the lesion with abundant blood supply, so the diagnosis of PGL was made.

Surgical resection is the standard treatment for PPGL, and the surgical plan depends on the location, diameter, blood supply, and surrounding tissues of the tumor. Due to the persistently high levels of CA, preoperative preparation with an α-receptor-blocker (such as Cardura) for at least 2 weeks is definitely necessary (23, 24). This kind of drug aims to block CA’s effect, maintain normal blood pressure and heart rate, and reduce operative complications that are associated with CA release. Intraoperative paroxysmal hypertension and severe blood pressure fluctuation may occur. For patients with metastatic PPGL, cytoreductive surgery and systemic therapy are considered. Surgery can not only relieve symptoms but also improve the efficacy of other treatments (25). Peptide receptor radionuclide therapy, such as Lu-177-DATATATE, is recommended for metastatic or inoperable PPGL (26). It has a disease control rate of 63.6% and was well tolerated in PPGL patients. In a phase 2 trial of Lu-177-DATATATE in 36 PPGL patients, the average progression-free survival (PFS) of patients with SDHx mutation is 15.4 months, and the PFS of patients in the sporadic cohort can reach 22.7 months (27). Chemotherapy, radiotherapy, and targeted therapy are optional therapy (8).

The recurrence rate of sporadic PPGL is approximately 15%. Patients with gene mutation, younger age, PGL, or larger tumor size have a higher risk of recurrence (28, 29). The prevalence of recurrence in patients with pseudohypoxic-related gene mutation is as high as 45% (29). PPGL patients need to be closely followed up, which includes clinical symptoms, plasma and urine fractionated MNs, 24-h urine CA, CT, and PET-CT scanning. Annual follow-up for at least 10 years after surgery is recommended (30). In this case, since the missense mutation of SDHA was detected, regular follow-up is necessary.

Spermatic cord PGL is rarer. The possible cause of this disease is the residual chromaffin cells in the embryo (31). To date, only 16 cases of spermatic cord PGLs have been reported (**Table 1**). Most of them did not show hormone-related symptoms and visited the doctor for a palpable scrotal mass. The diameter of their tumors ranged from 1.5 to 10 cm, with an average of 2.5 cm. Two of them have metastasis disease; one case with multiple metastases showed SDHB gene mutation, and another had spinal metastasis but no identified variants (32, 33). However, due to the lack of imaging characteristics, almost all cases were diagnosed by intraoperative frozen pathology or postoperative paraffin pathology. Given the special location of the spermatic cord PGL, it is risky because touching the mass can cause severe hypertension and blood pressure fluctuations and even ventricular fibrillation. The preoperative diagnosis based on extensive imaging and biochemical examination is quite important. In this case, the abnormally elevated serum and urinary CA led us to suspect the existence of PPGL. Then, after ruling out adrenal occupation, Ga^68^ DOTATATE PET-CT was performed, and a mass with high DOTATATE uptake in the right scrotum was demonstrated. A series of other imaging tests confirmed our diagnosis. After an adequate preoperative preparation with α-blockade to prevent intraoperative hypertensive crisis and a close cooperation between the anesthesiologist and the surgeon, the mass was resected successfully, and the pathological diagnosis established our diagnosis.

**Table 1:** Clinical characteristics of 17 cases reported as paraganglioma in the scrotum.

Time	Age	Site	Location	Maximum diameter (cm)	Hormonal symptoms	Gene mutation	Recurrence or metastasis
1971	37	R	Spermatic cord	2.5	None	Not measured	None
1977	52	L	Spermatic cord	4.5	Intraoperative paroxysmal hypertension	Not measured	None
1990	18	R	Spermatic cord	–	None	Not measured	None
1993	37	R	Spermatic cord	10	None	Not measured	None
1996	40	L	Spermatic cord	1.5	None	Not measured	None
1999	52	R	Spermatic cord	1.5	None	Not measured	None
2000	55	L	Spermatic cord	2	Intraoperative paroxysmal hypertension	Not measured	Recurrence of spermatic cord
2007	38	L	Spermatic cord	2.5	None	Not measured	None
2008	69	R	Spermatic cord	2	None	Not measured	None
2009	66	R	Spermatic cord	3.5	None	Not measured	None
2010	45	L	Spermatic cord	4.8	None	SDHD	None
2016	40	L	Spermatic cord	1.8	None	Not measured	None
2019	55	L	Spermatic cord	2.5	Palpitations	Not measured	None
2019	28	R	Spermatic cord	3.5	Hypertension, paroxysmal palpitations, and hyperhidrosis	SDHB	Retroperitoneum lymph nodes and lung
2020	40	R	Spermatic cord	3	None	Normal	None
2020	40	R	Spermatic cord	2.5	None	Normal	Spinal metastasis
This case	15	R	Spermatic cord	4.0	Hypertension and palpitations	SDHA	None

## Data availability statement

The original contributions presented in the study are included in the article/**Supplementary Material**, further inquiries can be directed to the corresponding authors.

## Ethics statement

The studies involving humans were approved by The Ethics Committee of Ruijin Hospital, Shanghai Jiao Tong University School of Medicine. The studies were conducted in accordance with the local legislation and institutional requirements. The human samples used in this study were acquired as part of a previous study for which ethical approval was obtained. Written informed consent for participation was not required from the participants or the participants’ legal guardians/next of kin in accordance with the national legislation and institutional requirements. Written informed consent was obtained from the individual(s) for the publication of any potentially identifiable images or data included in this article.

## Author contributions

YH: Data curation, Formal analysis, Investigation, Methodology, Software, Validation, Visualization, Writing – original draft, Writing – review & editing. XL: Data curation, Investigation, Writing – original draft. JX: Investigation, Methodology, Software, Writing – review & editing. WH: Data curation, Methodology, Resources, Visualization, Writing – review & editing. CW: Conceptualization, Project administration, Writing – review & editing. FS: Conceptualization, Project administration, Supervision, Writing – review & editing.

## Supplementary material

Supplementary Figure 1 Testicular CT was performed 4 months **(A)** and 8 months **(B)** after operation.
