# Supplementary material for: Case report: Rare case of a preoperatively diagnosed spermatic cord paraganglioma and literature review
Source: Front Oncol. 2024 Apr 11;14:1373727. doi: 10.3389/fonc.2024.1373727 (PMC11047120; doi:10.3389/fonc.2024.1373727)
Supplement: Supplementary file 1 [file Table_1.docx]

Table S1. Genes included in the endocrine tumors panel.

| AIP | ALK | ARID1A | ARMC5 | ATM | ATP1A1 | ATP2B3 | ATRX |
| --- | --- | --- | --- | --- | --- | --- | --- |
| BRAF | CACNA1D | CDC73 | CDKN1B | CSDE1 | CTNNB1 | DAXX | DNMT3A |
| EGLN1 | EIF1AX | EPAS1 | EZH2 | GNAS | HRAS | KCNJ5 | KMT2A |
| KMT2D | KRAS | MAX | MEN1 | NF1 | PRKACA | PRKAR1A | PTCH1 |
| RB1 | RET | SDHA | SDHB | SDHC | SDHD | SETD2 | SF3B1 |
| TMEM127 | TP53 | TSC2 | USP8 | VHL | YY1 |  |  |
