# Supplementary figures and images for: Case report: Rare case of a preoperatively diagnosed spermatic cord paraganglioma and literature review
Source: Front Oncol. 2024 Apr 11;14:1373727. doi: 10.3389/fonc.2024.1373727 (PMC11047120; doi:10.3389/fonc.2024.1373727)

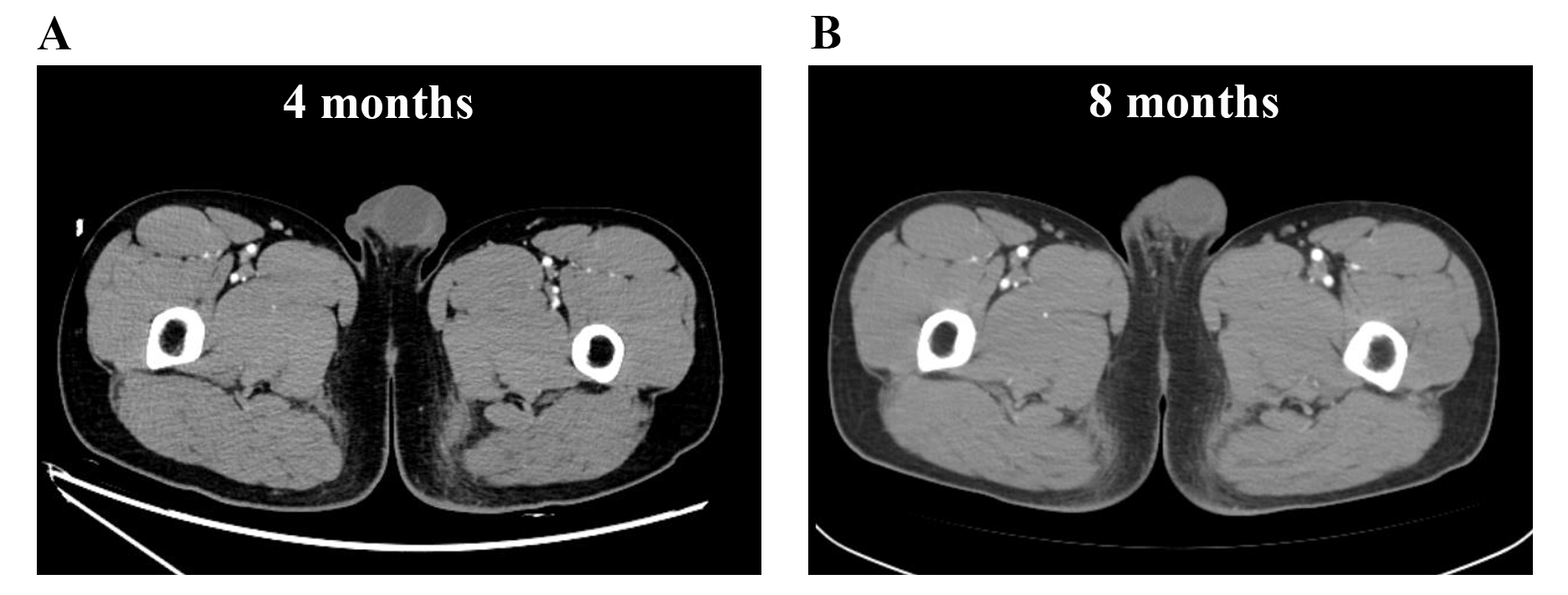

Supplement: Supplementary Figure 1 — Testicular CT was performed 4 months (A) and 8 months (B) after operation. [file Image_1.tif]
